# ERO1α is a novel endogenous marker of hypoxia in human cancer cell lines

**DOI:** 10.1186/s12885-019-5727-9

**Published:** 2019-05-29

**Authors:** Norio Takei, Akihiro Yoneda, Marina Kosaka, Kaori Sakai-Sawada, Yasuaki Tamura

**Affiliations:** Department of Molecular Therapeutics, Center for Food and Medical Innovation, Institute for the Promotion of Business-Regional Collaboration, Hokkaido University, Kita-21 Nishi-11, Kita-ku, Sapporo, 001-0021 Japan

**Keywords:** ERO1α, Hypoxia, Biomarker, CA9, Cancer

## Abstract

**Background:**

Hypoxia is an important factor that contributes to tumour aggressiveness and correlates with poor prognosis and resistance to conventional therapy. Therefore, identifying hypoxic environments within tumours is extremely useful for understanding cancer biology and developing novel therapeutic strategies. Several studies have suggested that carbonic anhydrase 9 (CA9) is a reliable biomarker of hypoxia and a potential therapeutic target, while pimonidazole has been identified as an exogenous hypoxia marker. However, other studies have suggested that CA9 expression is not directly induced by hypoxia and it is not expressed in all types of tumours. Thus, in this study, we focused on endoplasmic reticulum disulphide oxidase 1α (ERO1α), a protein that localises in the endoplasmic reticulum and is involved in the formation of disulphide bonds in proteins, to determine whether it could serve as a potential tumour-hypoxia biomarker.

**Methods:**

Using quantitative real-time polymerase chain reaction, we analysed the mRNA expression of ERO1α and CA9 in different normal and cancer cell lines. We also determined the protein expression levels of ERO1α and CA9 in these cell lines by western blotting. We then investigated the hypoxia-inducible ERO1α and CA9 expression and localisation in HCT116 and HeLa cells, which express low (CA9-low) and high (CA9-high) levels of CA9, respectively*.* A comparative analysis was performed using pimonidazole, an exogenous hypoxic marker, as a positive control. The expression and localisation of ERO1α and CA9 in tumour spheres during hypoxia were analysed by a tumour sphere formation assay. Finally, we used a mouse model to investigate the localisation of ERO1α and CA9 in tumour xenografts using several cell lines.

**Results:**

We found that ERO1α expression increased under chronic hypoxia. Our results show that ERO1α was hypoxia-induced in all the tested cancer cell lines. Furthermore, in the comparative analysis using CA9 and pimonidazole, ERO1α had a similar localisation to pimonidazole in both CA9-low and CA9-high cell lines.

**Conclusion:**

ERO1α can serve as a novel endogenous chronic hypoxia marker that is more reliable than CA9 and can be used as a diagnostic biomarker and therapeutic target for cancer.

**Electronic supplementary material:**

The online version of this article (10.1186/s12885-019-5727-9) contains supplementary material, which is available to authorized users.

## Background

Malignant tumours are characterised by a hypoxic environment, caused by an imbalance between the growth of the tumour cells and that of tumour-associated blood vessels [1]. The structural difference between tumour and normal blood vessels and the hypoxic microenvironment in tumours concur with the resistance to anticancer drugs and radiation [2]. Studies have shown that the hypoxic environment exacerbates the ability of cancer cells to migrate and invade, consequently promoting metastasis [3–5]. Therefore, to defeat cancer, it is essential to comprehend the mechanisms leading to cancer hypoxia entirely. Moreover, accurate detection of the hypoxic environment is a robust and useful tool to improve the understanding of cancer hypoxia.

Carbonic anhydrase 9 (CA9) and glucose transporter 1 (GLUT-1) are upregulated under hypoxic conditions through the upregulation and stabilisation of hypoxia-inducible factor 1 (HIF-1) and are therefore considered hypoxia markers [6–8]. Additionally, pimonidazole, administered exogenously and successively stained, is also considered a useful marker for the identification of low-oxygen environments [9]. Some studies, however, indicate that the expression of CA9 and GLUT-1 varies in different cancer cell lines [10, 11]. Additionally, immunohistochemistry for these markers in relation to pimonidazole staining has yielded conflicting results [12, 13]. Specifically, CA9 and GLUT-1 often lead to false negatives, arising from fluctuation errors, in patient specimens. Therefore, from a biological plausibility standpoint, CA9 and GLUT-1 cannot be considered accurate hypoxia biomarkers [14, 15]. In contrast, pimonidazole is more specific for hypoxia [16]; however, because it needs to be pre-administered, it requires a moderate invasive procedure and cannot be used in retrospective studies. It has been hypothesised that hypoxia-inducible molecules, such as HIF-1α, glucose transporters, and CA9, can be therapeutic targets [17]. This possibility, however, has not been thoroughly investigated and has not led to the establishment of a cure for cancer. Therefore, it is necessary to identify hypoxia biomarkers that are precise and can be used retrospectively, for example, in pre-existing paraffin sections, without the need for the administration of a substrate prior to tumour removal.

We have previously reported that the expression of endoplasmic reticulum disulphide oxidase 1α (ERO1α), an oxidase localized in the endoplasmic reticulum that plays a role in the formation of disulphide bonds, is higher in various types of cancer when compared to normal tissues, and that the expression levels of this molecule are associated with cancer progression and prognosis [18–21]. In addition, some studies have suggested that ERO1α is upregulated under hypoxia in cancer cell lines and tumours [22–26].

Therefore, in this study, we extensively investigated the expression of ERO1α and found that it is ubiquitously expressed in cancer cells, albeit at relatively modest levels, and is considerably induced under hypoxia. Furthermore, we inspected its utility as a tumour hypoxia marker by comparing its expression to that of CA9 using in vitro cell cultures and an in vivo xenograft model.

## Methods

### Cell culture

Normal cell lines (NHDF, Lonza® CC-2511; HEK293, ATCC® CRL-1573) and cancer cell lines (colon: HCT116, ATCC® CCL-247; SW480, ATCC® CCL-228; HT-29, ATCC® HTB-38; breast: MDA-MB-231, ATCC® HTB-26; MCF7, ATCC® HTB-22; cervical: HeLa, ATCC® CCL-2; lung: A549, ATCC® CCL-185; pancreatic: MIAPaCa2, ATCC® CRL-1420; PANC1, ATCC® CRL-1469; liver: HepG2, ATCC® CRL-10741; fibrosarcoma: HT-1080, ATCC® CCL-121), were obtained from Lonza Inc. (Walkersville, MD, USA) and American Type Culture Collection (ATCC, Manassas, VA, USA). These cell lines are not listed in the database of the International Cell Line Authentication Committee (ICLAC). The cells were cultured in DMEM (Sigma-Aldrich, St. Louis, MO, USA) supplemented with 10% foetal bovine serum (FBS; Gibco, Gaithersburg, MD, USA) and 100 U/ml of penicillin and streptomycin and routinely tested for mycoplasma contamination. All cells were incubated at 37 °C in a humidified atmosphere containing 95% air and 5% CO_2_. For testing the effects of hypoxia, cells were cultured in a hypoxia CO_2_ incubator (1% O_2_/94% N_2_/5% CO_2_) for different time intervals. At each time point, cells were collected for quantitative PCR (qPCR) and western blotting (WB) using a scraper. Acute hypoxia was defined as culture under hypoxic conditions for less than 24 h, while chronic hypoxia referred to hypoxic culture for over 24 h [27, 28]. RNA was purified using the RNeasy Mini kit (QIAGEN, Germantown, MD, USA) and protein samples were obtained using TNE lysis buffer (1% Nonidet P-40; 50 mM Tris-HCl, pH 8.0; 150 mM NaCl; 25 mM EDTA; 100 mM NaCl) or RIPA buffer (50 mM Tris, pH 8.0; 150 mM NaCl; 0.5% deoxycholate; 1% Nonidet P-40; 0.1% sodium dodecyl sulphate [SDS]) containing a protease inhibitor cocktail (Roche Diagnostics, Basel, Switzerland).

The cells were incubated for 2 h in the presence of 100 μM Hypoxyprobe™-1 (Hypoxyprobe, Inc., Burlington, MA, USA) for pimonidazole binding. Cells were cultured using Costar® 6-Well Clear Flat Bottom Ultra Low Attachment Multiple Well Plates (Corning, Corning, NY, USA) for the sphere formation assay. Aggregates of 150–200 μm in diameter were collected and embedded with iPGell (GenoStaff, Tokyo, Japan) and sectioned, formalin-fixed, paraffin-embedded samples were subjected to immunohistochemistry.

### Xenograft model

All animal studies were conducted in compliance with the guidelines of the Animal Studies Committee of Hokkaido University (Approval Number #15–0170). BALB/c nude mice (6–8 weeks old) were obtained from CLEA (Tokyo, Japan), kept on a 12 h light/12 h dark schedule (lights on from 7:00 to 19:00) and allowed food and water ad libitum. For tumour-formation studies, mice were injected with 1 × 10^6^ cells of a specific cell line in the lateral subcutaneous region. The cells lines used were HCT116, HeLa, A549, and MIAPaCa2 (*n* = 4). 28 days after transplantation, pimonidazole (60 mg/kg body weight [bw]) was administered through the tail vein. Mice were euthanised with sodium pentobarbital (120 mg/kg [bw], intraperitoneally) 1 h after the treatment and tumours were excised and stored in 10% formalin for histological analysis.

### Quantitative PCR

Total RNA was isolated from the cells using RNeasy Mini kits (QIAGEN) according to the manufacturer’s protocol, and cDNA was synthesised from 500 ng of total RNA using the SuperScript® VILO™ cDNA Synthesis Kit (Thermo Fisher Scientific, Waltham, MA, USA). The expression of *ERO1A* and *CA9* mRNA was analysed by qPCR, performed using an Applied Biosystems 7500 Fast Real-Time PCR system and PowerUp™ SYBR® Green Master Mix (Applied Biosystems, Carlsbad, CA, USA), according to the manufacturer’s recommendations. PCR was performed in 96-well plates as follows: denaturation at 95 °C for 20 s, followed by 40 cycles of annealing at 95 °C for 3 s and amplification at 60 °C for 30 s. Each sample was run in triplicate. Quantification relative to *ERO1A* and *CA9* mRNA expression in normoxic cultures was performed using the comparative Ct method [29] using the expression of the actin B gene (*ACTB)* as an internal control. The sets of primers used in this study had been previously described [30, 31].

### Western blot analysis

Cells grown in cultures were washed twice with phosphate-buffered saline (PBS), followed by scraping and collection using lysis buffer (TNE or RIPA) containing phosphatase and protease inhibitors (Roche Diagnostics). The lysates were centrifuged, and the collected supernatants were used for western blotting (WB), which was performed as previously described [24, 32]. Briefly, cell lysates were separated by SDS-polyacrylamide gel electrophoresis (SDS-PAGE) on SuperSep Ace gels (Wako, Tokyo, Japan) and transferred to polyvinylidene difluoride (PVDF) membranes (Millipore, Temecula, CA, USA) using standard techniques and instruments (Bio-Rad, Irvine, CA, USA). The immobilised proteins were blocked in sterilised 0.5% non-fat dry milk dissolved in PBS containing 0.05% Tween-20 (PBST) and then incubated overnight at 4 °C with primary polyclonal antibodies. The primary antibodies used were as follows: anti-ERO1α (Abnova, Taipei City, Taiwan), anti-CA9 (Abcam, Cambridge, MA, USA), anti-HIF-1α (BD Biosciences, San Jose, CA, USA), anti-HIF-2α (Novus Biologicals, Littleton, CO, USA), anti-pimonidazole (Hypoxyprobe, Inc.), anti-glyceraldehyde 3-phosphate dehydrogenase (GAPDH), and anti-β-actin (Abcam). The membranes were washed and incubated with a secondary antibody (horseradish peroxidase [HRP]-conjugated goat anti-mouse or anti-rabbit antibodies; Cell Signaling Technology, Danvers, MA), according to the manufacturer’s instructions, and then washed repeatedly with PBST before visualization of the immunoreactive complexes using ECL reagent (Thermo Fisher Scientific) in a Chemidoc Imaging System (Bio-Rad). All experiments were repeated thrice. The intensities of the protein bands of ERO1α and CA9 were normalised to the values of GAPDH.

### Immunohistochemistry

Formalin-fixed, paraffin-embedded xenograft tumour samples were sectioned, deparaffinized, and incubated in histofine agent retrieval buffer, pH 9 (Nichirei Biosciences, Tokyo, Japan) at 105 °C for 15 min. After washing and blocking of non-specific antibody-binding sites, each section was incubated with mouse anti-human ERO1α, rabbit anti-pimonidazole (both of which are described above), or anti-CA9 (ab15086, Abcam) primary antibodies overnight at 4 °C, followed by incubation with anti-mouse or anti-rabbit IgG (H + L) goat IgG Fab’-HRP secondary antibodies (IBL, Gunma, Japan) for 45 min, and subsequently developed using 3,3α-diaminobenzidine tetrachloride (DAB) for bright-field microscopy.

For immunofluorescence imaging, the sections were incubated with primary antibodies at 4 °C, overnight, washed with PBS, and then incubated with AlexaFluor-488-conjugated goat anti-rabbit IgG or AlexaFluor-555-conjugated goat anti-mouse IgG secondary antibodies (Life Technologies, Camarillo, CA, USA). The specimens were mounted with ProLong Diamond Antifade Mountant with DAPI (Thermo Fisher Scientific) and observed using a fluorescence microscope; the same imaging conditions were used for all samples.

### Statistical analysis

The data showed are representative of at least three independent experiments unless stated otherwise and are reported as the mean ± the standard error of the mean (SEM). Differences among groups were analysed using the Student’s t-test. We performed analysis of variance (ANOVA) followed by the Bonferroni test to compare more than two groups. *P* < 0.05 was considered statistically significant.

## Results

### ERO1α and CA9 expression in various cell lines

**Fig. 1:**
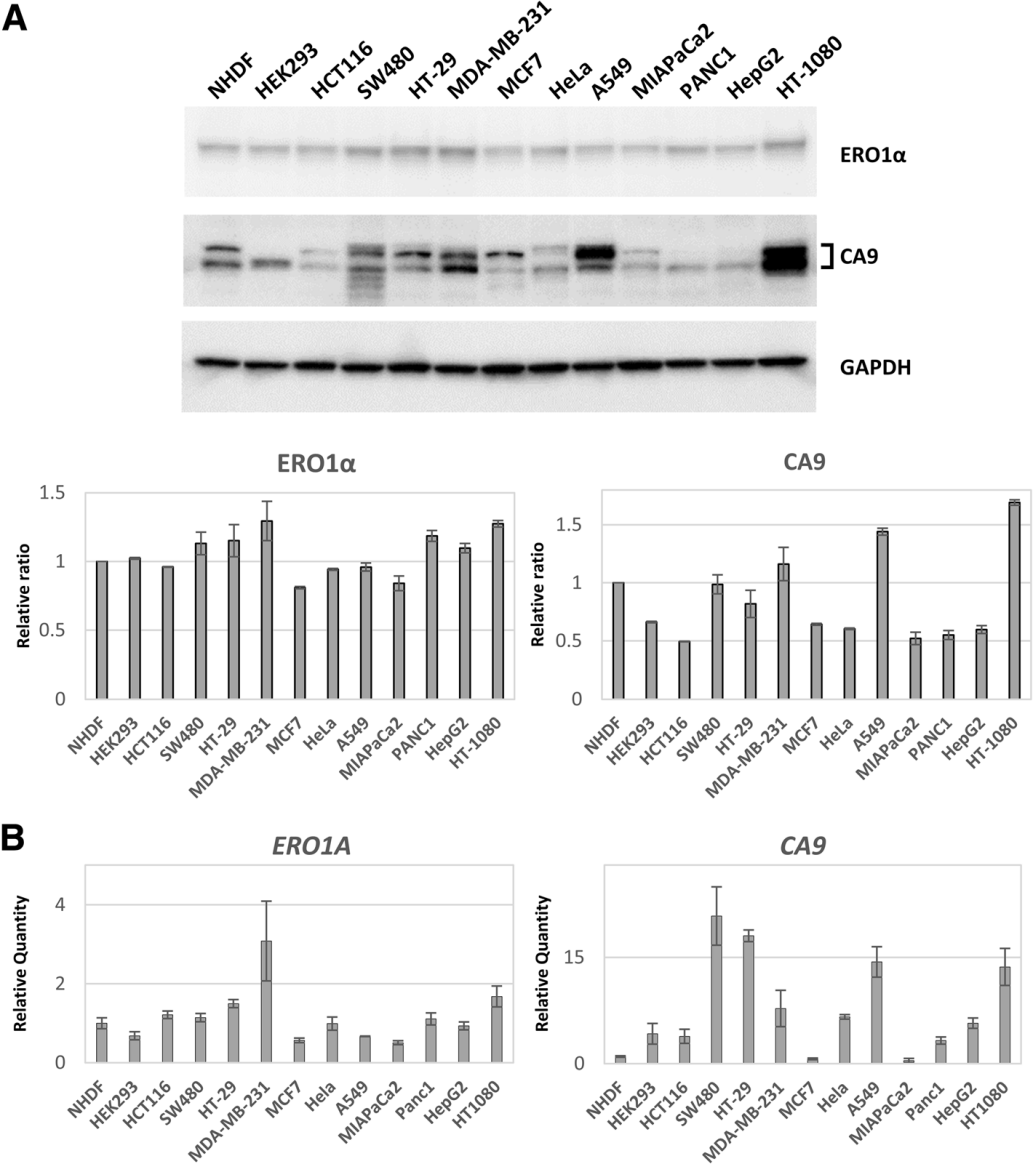
Expression of ERO1α and CA9 in human cell lines. **a** Endoplasmic reticulum disulphide oxidase 1α (ERO1α) and carbonic anhydrase 9 (CA9) protein expression in normal (NHDF and HEK293) cell lines and cells lines from different cancer types: colon (HCT116, SW480, and HT-29); breast (MDA-MB-231 and MCF7); cervical (HeLa); lung (A549); pancreatic (MIAPaCa2 and PANC1); liver (HepG2); and fibrosarcoma (HT-1080). The cells were analysed by western blotting (WB). Quantified values of ERO1α (left graph) and CA9 (right graph) are indicated. The values were normalised to those of glyceraldehyde 3-phosphate dehydrogenase (GAPDH). The values obtained from NHDFcells were set to 1.0 and are given as the mean ± the standard error of the mean (SEM) (**b**) Gene expression analysis of ERO1α and CA9 by quantitative PCR (qPCR) in the above-mentioned cell lines. The relative amounts of mRNA were calculated from the comparative threshold cycle (Ct) values relative to those of GAPDH. Data are presented as the mean ± the standard error of the mean (SEM)

First, we confirmed the mRNA and protein expression of ERO1α and CA9 in various normal and cancer cell lines under normoxic in vitro culture by qPCR and WB analyses. We found that the protein and mRNA expression patterns of ERO1α mostly correlated well with each other (Fig. 1a and b). Additionally, under normoxia, ERO1α was expressed in all cell lines, though with some variations, and was relatively more abundant in the cancer cell lines than in the normal cell lines. In contrast, the protein expression of CA9 in the cell lines analysed was not ubiquitous and did not correlate well with its mRNA expression.

### ERO1α expression is enhanced in hypoxic conditions

**Fig. 2:**
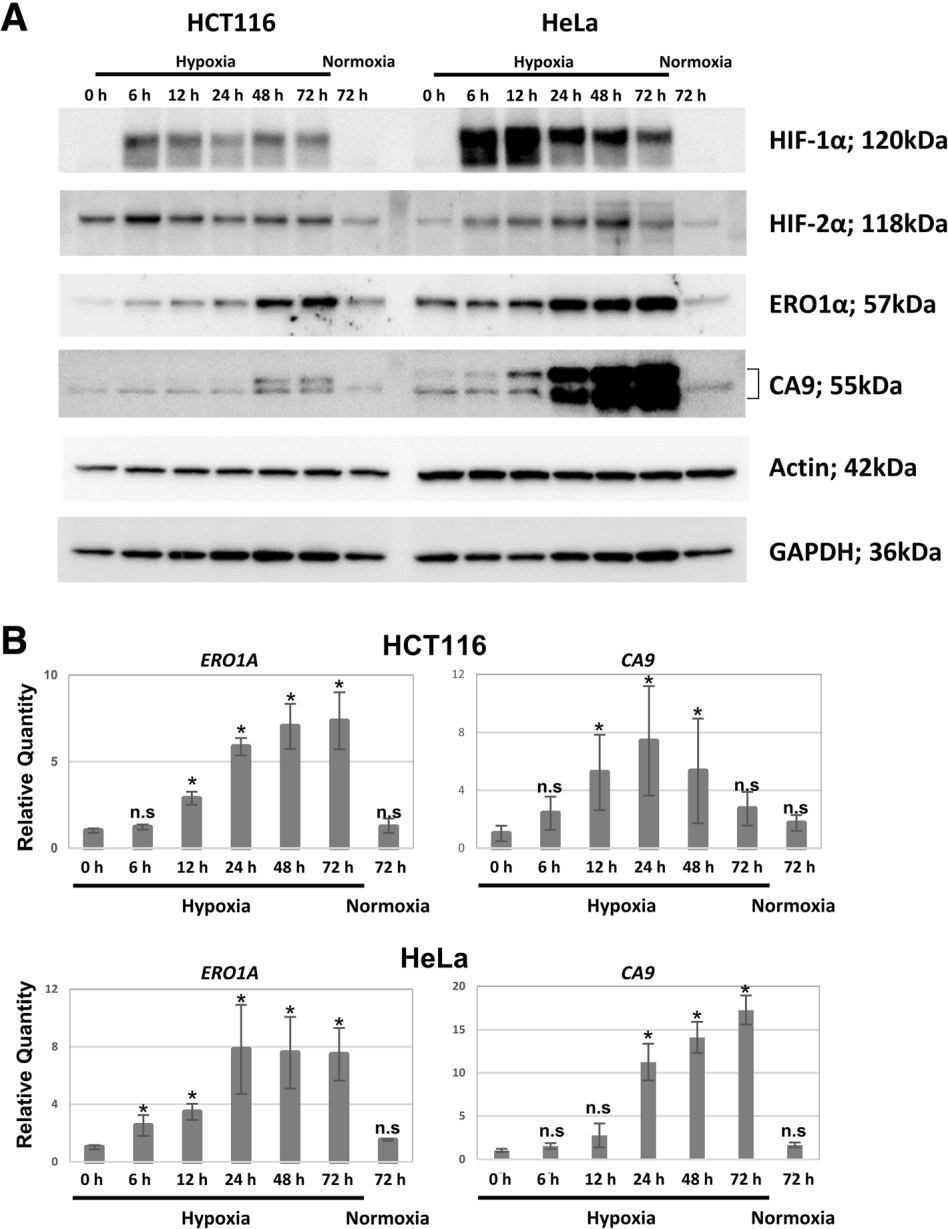
ERO1α and CA9 expression profiles in HCT116 and HeLa cells under normoxia and hypoxia. The expression of ERO1α and CA9 at the protein (**a**) and mRNA (**b**) levels were investigated over time in hypoxic cultures by western blotting (WB) and quantitative PCR (qPCR), respectively, using HCT116 cells (CA9-low) and HeLa cells (CA9-high). Cultured cells were incubated for 0, 6, 12, 24, 48, or 72 h under hypoxia. Cells incubated for 72 h under normoxia were used as a control. In WB analysis, β-actin and glyceraldehyde 3-phosphate dehydrogenase (GAPDH) were used as internal controls. HIF1α was detected to confirm hypoxia. In the qPCR analysis, the relative mRNA levels were calculated from the comparative threshold cycle (Ct) values relative to the actin B gene (*ACTB*). Data are presented as the mean ± the standard error of the mean (SEM, *n* = 4). *P < 0.05 calculated by ANOVA followed by a Bonferroni test. n.s.: not significant

We focused on the HCT116 and HeLa cell lines, which have low (CA9-low) and high levels (CA9-high) of hypoxia-induced CA9, respectively, and measured the changes in ERO1α and CA9 protein expression in cells grown under normal (normoxia) and low (hypoxia) oxygen concentration by WB. As shown in Fig. 2a, the expression of the two members of the HIF family HIF-1α and HIF-2α increased under hypoxic condition. HIF-1α expression peaked 6 h after exposure to low oxygen concentrations and decreased afterwards in both cell lines. HIF-2α behaved similarly to HIF-1α in HCT 116 cells, while in HeLa cells it gradually increased in hypoxic conditions, and peaked 48 h after exposure to low oxygen concentrations.

ERO1α and CA9 expression gradually increased in a time-dependent manner during the entire observation period in the cells cultured under hypoxic conditions. Specifically, the levels of ERO1α increased in both cell lines, whereas the expression of CA9 was remarkably enhanced in HeLa cells and, in contrast, remained low in HCT116 cells. Our results with HCT116 cells showed that the expression patterns of HIF1α and CA9 were different which is not consistent with a previous study indicating that HIF-1α induces the expression of CA9 [33]. Hence, it may not be concluded that CA9 is a surrogate marker of HIF-1α.

To confirm the expression at the mRNA level of ERO1α and CA9 in a hypoxic environment, we evaluated the changes in the mRNA levels of these molecules under hypoxia by qPCR (Fig. 2b) and found that in HeLa cells, the expression of both ERO1α and CA9 increased in a time-dependent manner under hypoxic conditions (Fig. 2b lower panels). Additionally, the upregulation of CA9 was significantly higher than that of ERO1α. The expression of ERO1α in HCT116 followed a similar trend, whereas the expression of CA9 increased during the first 24 h of incubation in hypoxic conditions and decreased afterwards until reaching levels similar to those of cells cultured in normoxia after 72 h (Fig. 2b upper panels). We verified the hypoxia-inducibility at the mRNA level using the other 6 cell lines and found that ERO1A showed significant hypoxia-inducibility in all cell lines, whereas CA9 showed no significant inducibility in the MCF7, MIAPaCa2, and Panc1 cell lines. (Additional file 1: Figure S1A).

### Immunohistochemical analysis of ERO1α and CA9 expression during hypoxia

**Fig. 3:**
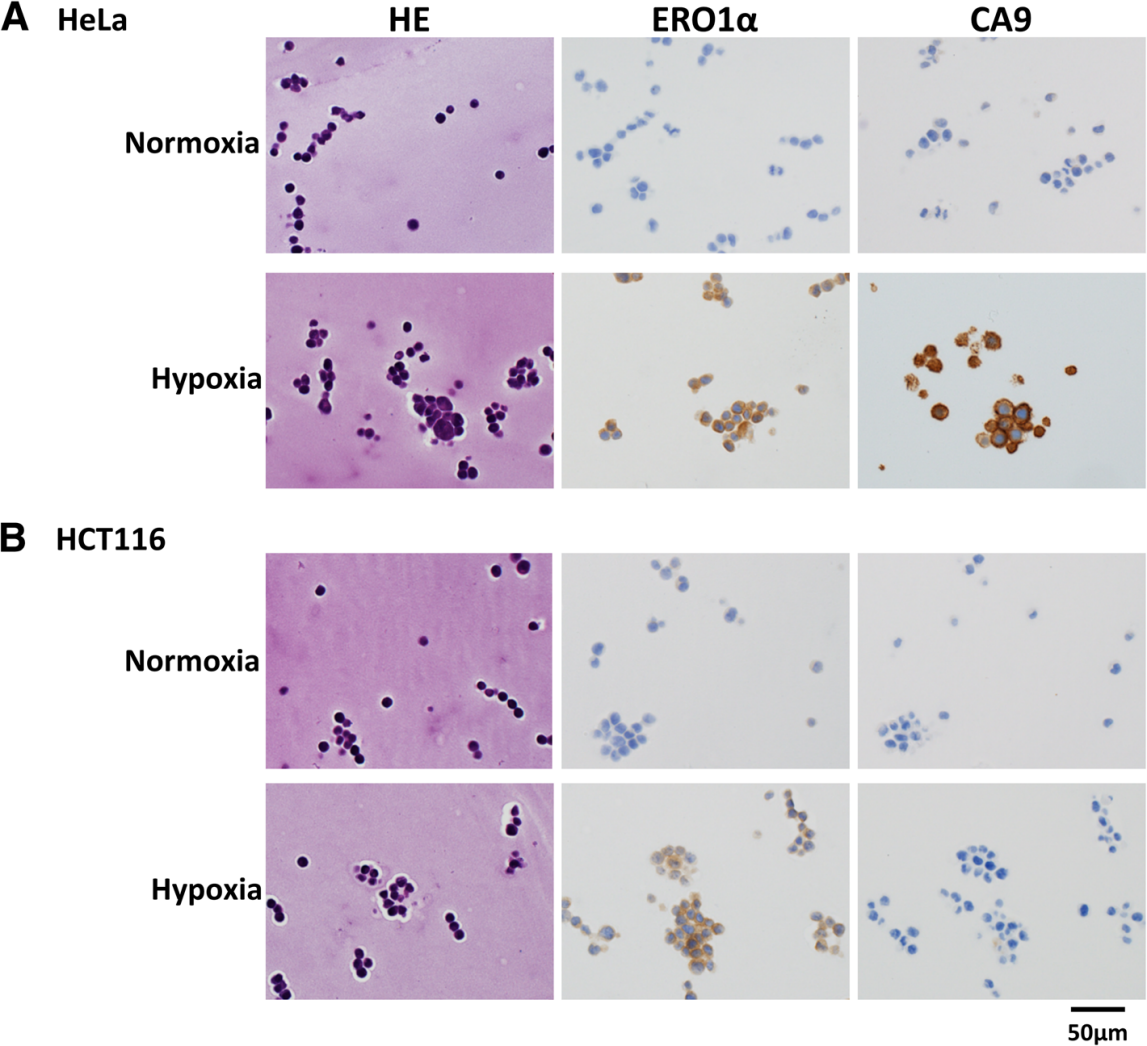
Immunohistochemical analysis of ERO1α and CA9 in cultured cells. HeLa (**a**) or HCT116 (**b**) cells were cultured until sub-confluent, collected using iPGell, formalin-fixed, paraffin-sectioned, and stained. Representative haematoxylin and eosin (HE, left), ERO1α (middle), and CA9 (right) staining images of cells grown under normoxia (upper panel) and hypoxia (lower panel) are shown. Scale bar: 50 μm

We next confirmed the WB data relative to the expression of ERO1α and CA9 by immunohistochemistry, which is routinely used as an evaluation method during pathological diagnosis. We used specific antibodies to identify ERO1α and CA9 in formalin-fixed, paraffin-embedded samples from cells cultured in normal and hypoxic conditions. As shown in Fig. 3, in HeLa cells, hypoxia led to an increase in the number of ERO1α- and CA9-positive cells. In the HCT116 cell line, there were few CA9-positive cells, regardless of the cell culture conditions whereas ERO1α-positive cells were clearly identified after hypoxia-incubation.

### Immunohistochemical analysis of ERO1α and CA9 expression using tumour spheres

**Fig. 4:**
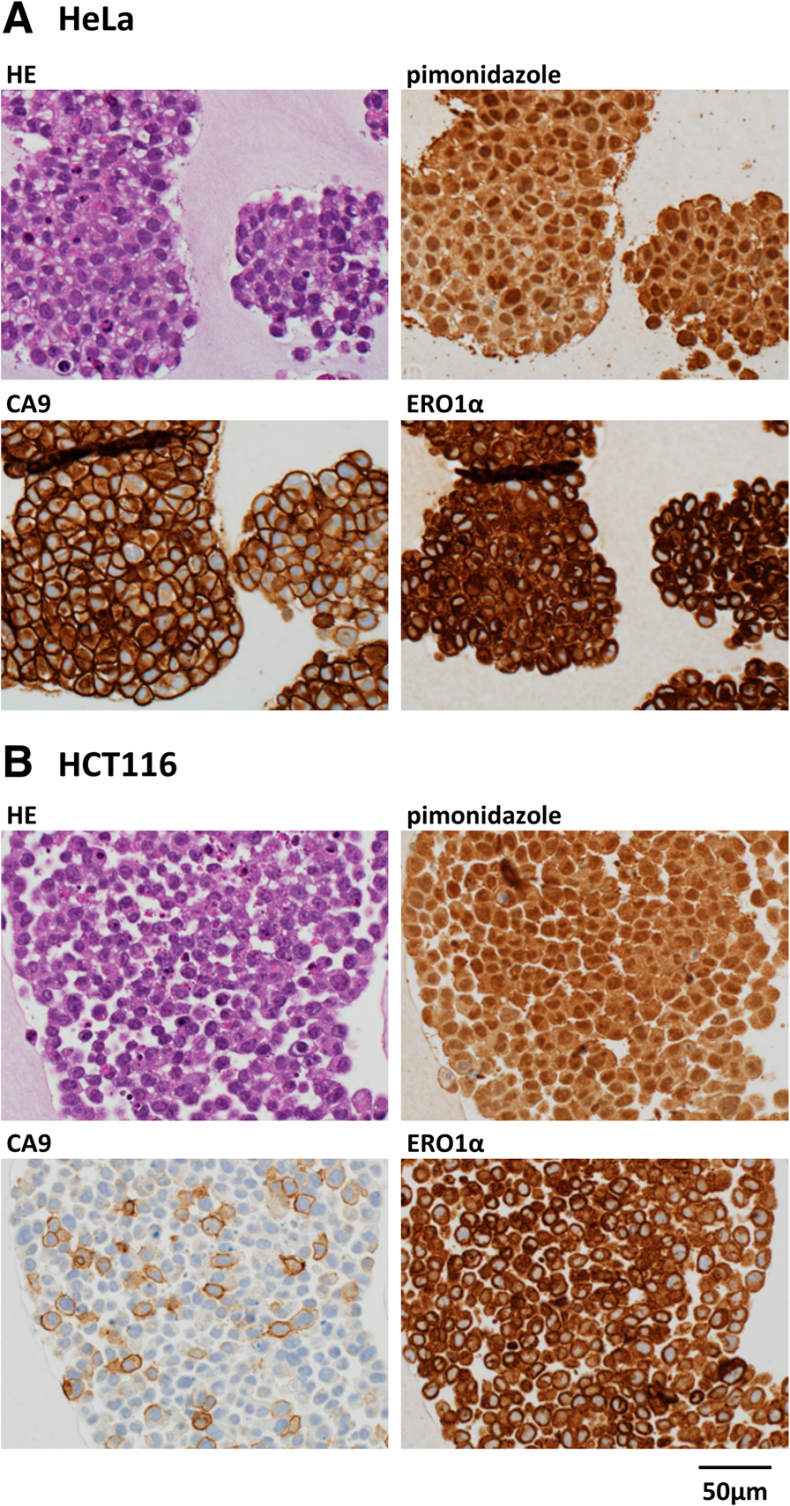
Expression of ERO1 and CA9 in tumour spheres from HCT116 and HeLa cells. Immunostaining using anti-pimonidazole, anti-ERO1α, and anti-CA9 antibodies in spheres obtained using Ultra low attachment plates as described in the Materials and Methods. **a** HeLa cells; **b** HTC116 cells. Representative tissue photographs are shown. Scale bar: 50 μm

Since tumour spheres simulate the actual tumour hypoxic microenvironment, we analysed the expression and localisation of ERO1α and CA9 in tumour spheres obtained from HCT116 and HeLa cells through sphere formation assays. Pimonidazole, the exogenous hypoxia marker described above, was used as a control. We found that, in spheres obtained from HeLa cells, ERO1α- and CA9-positive cells were also pimonidazole-positive (Fig. 4a). In contrast, in spheres obtained from HCT116 cells, the expression of CA9 could hardly be detected, and cells that were positive for pimonidazole and ERO1α were mostly negative for CA9 (Fig. 4b).

### ERO1α and CA9 localisation in HCT116 tumour xenografts

**Fig. 5:**
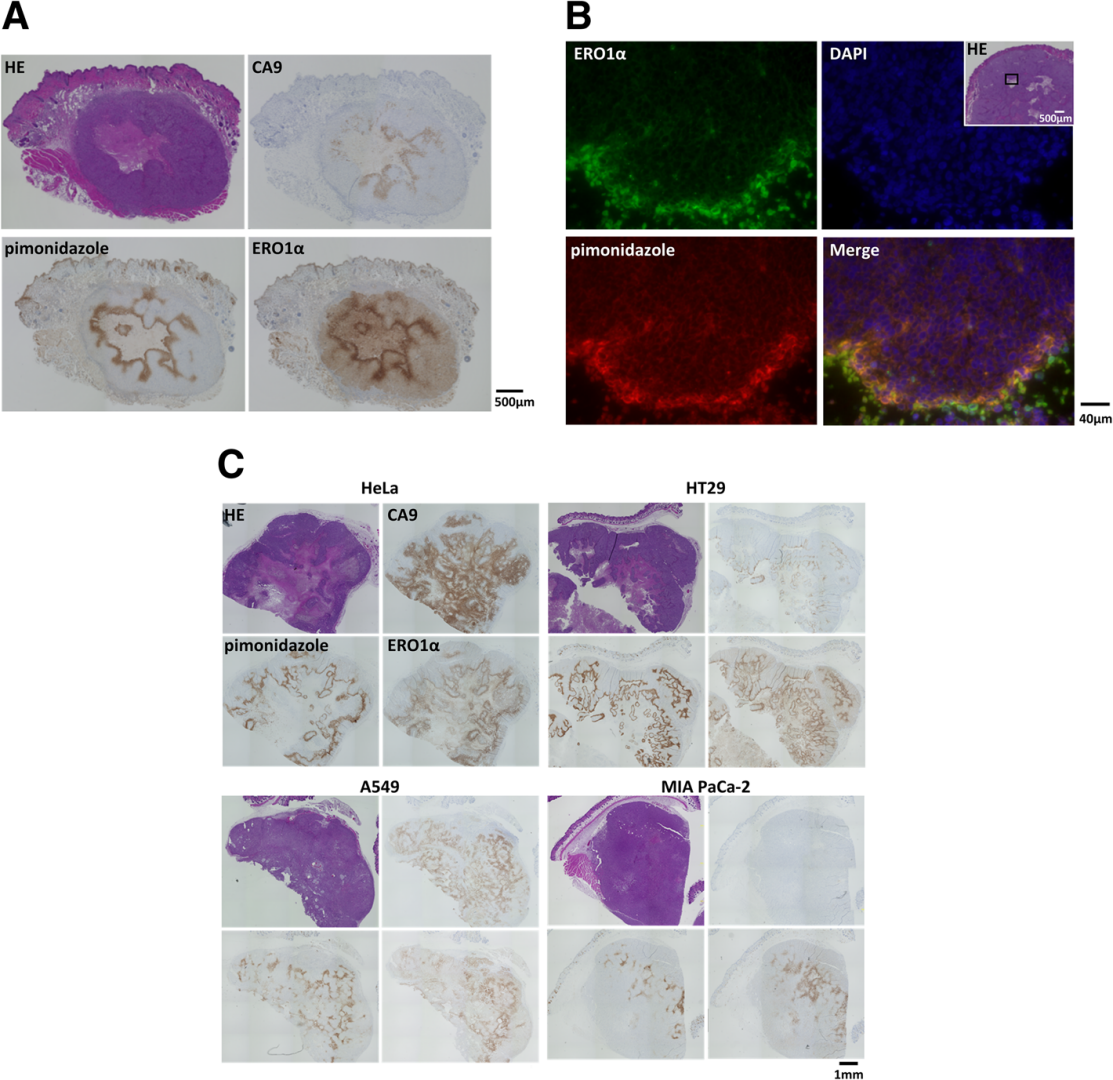
Colocalisation of ERO1α and pimonidazole at hypoxic sites in tumour xenografts. HCT116 cells were inoculated into nude mice; 28 days later, pimonidazole was intravenously injected. Tumours (*n* = 4) were excised and subjected to immunostaining. Representative images are shown. **a** Immunohistological staining using 3,3′-diaminobenzidine (DAB) as chromogenic substrate. Scale bar: 500 μm (**b**) Fluorescent immunostaining of ERO1α (green) and pimonidazole (red). 4′,6-Diamidino-2-phenylindole (DAPI, blue) was used to localise the nuclei. Scale bar: 40 μm. **c** Localisation of ERO1α, pimonidazole, and CA9 in various cancer cell line (HeLa, HT29, A549, and MIA PaCa2) tumour xenografts. Representative tissue photographs are shown. Scale bar: 1 mm

HCT116 cells were transplanted into nude mice to compare the localisation of ERO1α and CA9 in in vivo tumours. The resulting tumour xenografts were subsequently analysed by immunohistochemistry using antibodies specific for ERO1α and CA9, and the localisation of the proteins was compared to the staining with pimonidazole. We found an overlap between the pimonidazole- and the ERO1α-immunoreactive regions, while the staining specific for CA9 was weaker and did not overlap with that of pimonidazole (Fig. 5a).

We subsequently investigated the possible co-localisation of ERO1α and pimonidazole by immunofluorescence staining and observed a strong and largely overlapping fluorescent signal for ERO1α and pimonidazole in the central necrotic part of the tumour (Fig. 5b). In other cell lines, the expression of ERO1α was ubiquitously induced by hypoxia and showed a localisation similar to that of pimonidazole, whereas the expression of CA9 was inconsistent in the MIAPaCa2 and HT-29 cells lines (Fig. 5c).

## Discussion

We investigated whether ERO1α, a protein that contributes to the oxidative folding of molecules involved in cancer progression, can be considered a novel, endogenous hypoxia marker. We analysed changes over time in its mRNA and protein levels under normoxia and hypoxia culture conditions using cancer cell lines. Additionally, we compared ERO1α’s versatility to that of accepted endogenous (CA9) and exogenous (pimonidazole) hypoxia markers. Specifically, CA9 is considered a useful hypoxic and tumour marker and a “surrogate” of HIF-1α because its expression is induced by HIF1-α.

Our results indicate that the expression of CA9 at the mRNA and protein level fluctuates considerably among different cell lines, some of which do not express the protein. Therefore, CA9 should not be used as a hypoxia marker in all cancer cell lines [34, 35]. In contrast, ERO1α was constitutively expressed at the mRNA and protein levels in all the cell lines investigated in this study and its expression increased under hypoxic conditions, indicating that it can be used as a hypoxic marker in a wide range of cancer cell types.

We focused on HCT116 and HeLa cells which are representative cell lines that express low and high levels of CA9 under hypoxia, respectively, and compared the induction of CA9 and ERO1α when cells were grown in hypoxic conditions. In particular, we focused on chronic hypoxia, which has been implicated as a causal factor for the increased aggressiveness of tumours. Based on previous reports [27, 28], we defined hypoxia as acute when cells were cultured for less than 24 h under hypoxic conditions and chronic when cells were in hypoxic culture for over 24 h.

ERO1α appeared to be hypoxia-inducible in both cell lines, and its expression was especially enhanced during chronic hypoxia. In contrast, we confirmed that the hypoxia-inducibility of CA9 in HCT116 cells was remarkably weaker than that in HeLa cells, the mRNA level peaked after 24 h of culture in hypoxic conditions and decreased afterwards until reaching levels similar to that of control cells after 72 h.

It is possible that CA9 is controlled directly by HIF-1α, as the CA9 promoter contains a single hypoxia-responsive element (HRE) which binds to HIF. It has been previously reported that the HRE is a central regulatory element in the transcriptional regulation of CA9 [36, 37]. HIF-1α is induced by relatively transient hypoxia [38]. However, unlike previous studies, it was suggested that the expression of CA9 reflects not only transient hypoxia, like its inducer HIF-1α but also chronic hypoxia [13, 39, 40]. It is conceivable that CA9 is regulated by the activation of Unfolded Protein Response, independent of HIF-1α induction [41]. Consequently, we believe that CA9 is not an optimal marker for the detection of tumour hypoxia, an important factor in cancer progression, while ERO1α can be a true hypoxic marker.

Our immunohistochemical studies with ERO1α, CA9, and pimonidazole (an exogenous marker typically used to detect chronic hypoxia in tumour xenografts) indicated that the distribution of pimonidazole and ERO1α were very similar. However, the staining of CA9 was very weak and localised to necrotic areas of the tumour, as previously reported [42]. Additionally, it did not colocalise with either pimonidazole or ERO1α. Therefore, ERO1α can be considered a novel hypoxia marker, more suitable than CA9.

The variability in CA9 expression observed in our in vitro and in vivo studies further supports the proposal that CA9 should not be used as a hypoxia marker during a cancer diagnosis. Conversely, ERO1α appears to be a reliable marker of chronic hypoxia because of its predictable expression across different cell lines and colocalisation with pimonidazole.

Interestingly, some studies indicate the correlation between the severity of cancer and ERO1α positivity [18, 43, 44]. Additional studies, including the analysis of various carcinomas in vitro and samples from patients, are necessary to establish the prognostic value of ERO1α. Nevertheless, our observations reinforce the importance and usefulness of ERO1α as a prognostic marker in cancer since hypoxia affects both cancer cells and the tumour microenvironment and plays a pivotal role in the process of cancer progression [45].

ERO1α is the only oxidoreductase that can be induced by hypoxia [19, 22–24]. It is possible that ERO1α functions predominantly in the structural formation of various molecules under hypoxic conditions, strengthening the causal relationship between increased expression of ERO1α and tumour progression.

## Conclusion

In summary, we found that (1) ERO1α is an endogenous hypoxia marker; specifically, a marker of chronic hypoxia; and (2) ERO1α is expressed under hypoxic conditions in cell lines with both high and low CA9 expression. Collectively, we believe that the expression of ERO1α is independent of the specific cancer cell line or cancer type and that ERO1α is a reliable marker for the identification of hypoxic tumour microenvironments. Further studies might highlight the role of ERO1α in the prediction of cancer prognosis and the effect of ERO1α-targeting for anti-cancer therapies.

## Additional file

Additional file 1: **Figure S1.** ERO1α and CA9 gene expression in cancer cell lines under normoxia and hypoxia. ERO1α and CA9 mRNA levels in hypoxic cultures were investigated by qPCR in various cancer cell lines. Cells were incubated for 72 h under normoxia or hypoxia. The level of mRNA under normoxic conditions was used as a control. The relative mRNA levels were calculated from the comparative threshold cycle (Ct) values relative to *ACTB*. Data are presented as the mean ± the standard error of the mean (SEM, *n* = 4). N: normoxia; Hy: hypoxia. (TIF 1826 kb)

## Abbreviations

ACTB: Actin B
bw: body weight
CA9: Carbonic anhydrase 9
DAB: 3,3α-diaminobenzidine tetrachloride
DAPI: 4′,6-diamidino-2-phenylindole
ERO1α: Endoplasmic reticulum disulphide oxidase 1α
FBS: Foetal bovine serum
GAPDH: Glyceraldehyde 3-phosphate dehydrogenase
GLUT-1: Glucose transporter 1
HIF-1: Hypoxia-inducible factor 1
HRP: Horseradish peroxidase
PBS: Phosphate-buffered saline
PBST: PBS containing 0.05% Tween-20
PVDF: Polyvinylidene difluoride
qPCR: quantitative PCR
SDS: Sodium dodecyl sulphate
SDS-PAGE: SDS-polyacrylamide gel electrophoresis
SEM: Standard error of the mean
WB: Western blotting
