# Supplementary figures and images for: ERO1α is a novel endogenous marker of hypoxia in human cancer cell lines
Source: BMC Cancer. 2019 May 29;19:510. doi: 10.1186/s12885-019-5727-9 (PMC6542132; doi:10.1186/s12885-019-5727-9)

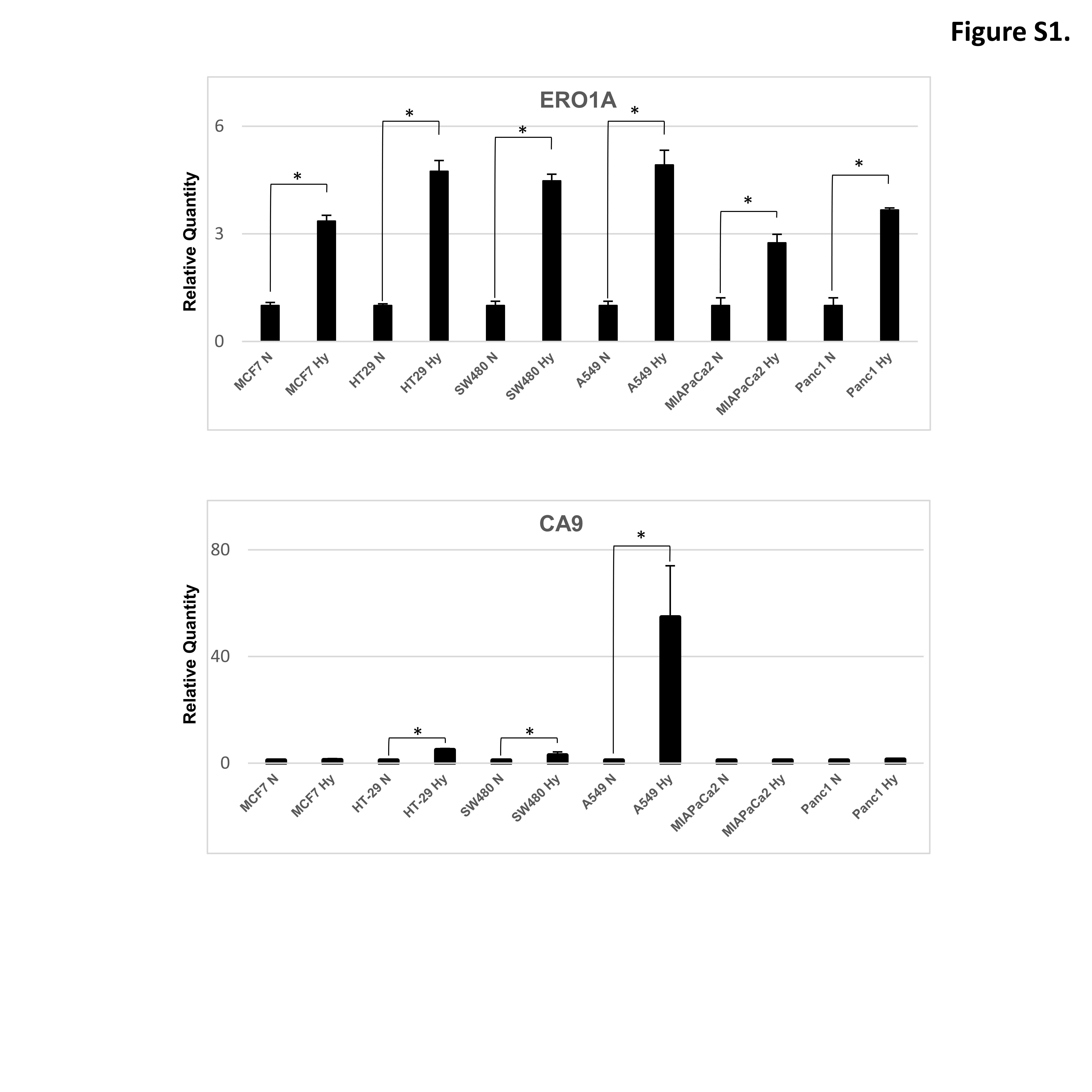

Supplement: Supplementary file 1 — Figure S1. ERO1α and CA9 gene expression in cancer cell lines under normoxia and hypoxia. ERO1α and CA9 mRNA levels in hypoxic cultures were investigated by qPCR in various cancer cell lines. Cells were incubated for 72 h under normoxia or hypoxia. The level of mRNA under normoxic conditions was used as a control. The relative mRNA levels were calculated from the comparative threshold cycle (Ct) values relative to ACTB. Data are presented as the mean ± the standard error of the mean (SEM, n = 4). N: normoxia; Hy: hypoxia. (TIF 1826 kb) [file 12885_2019_5727_MOESM1_ESM.tif]
